# Supplementary material for: Disentangling Abstraction from Statistical Pattern Matching in Human and Machine Learning
Source: PLoS Comput Biol. 2023 Aug 25;19(8):e1011316. doi: 10.1371/journal.pcbi.1011316 (PMC10497163; doi:10.1371/journal.pcbi.1011316)
Supplement: S1 Fig — (PDF) [file pcbi.1011316.s001.pdf]

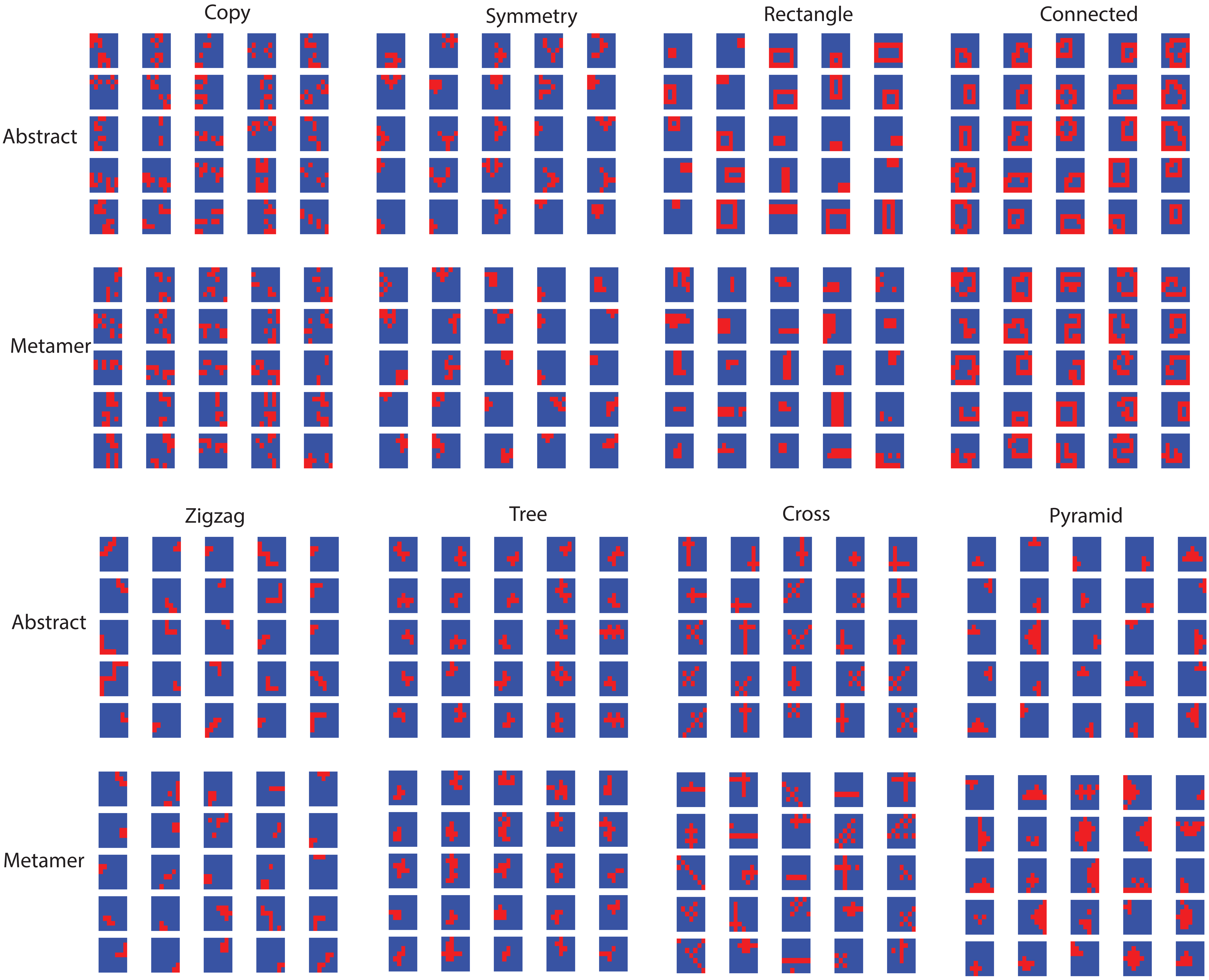
Copy
Symmetry
Rectangle
Connected
Abstract
Metamer
Zigzag
Tree
Cross
Pyramid
Abstract
Metamer
