# Supplementary figures and images for: Disentangling Abstraction from Statistical Pattern Matching in Human and Machine Learning
Source: PLoS Comput Biol. 2023 Aug 25;19(8):e1011316. doi: 10.1371/journal.pcbi.1011316 (PMC10497163; doi:10.1371/journal.pcbi.1011316)

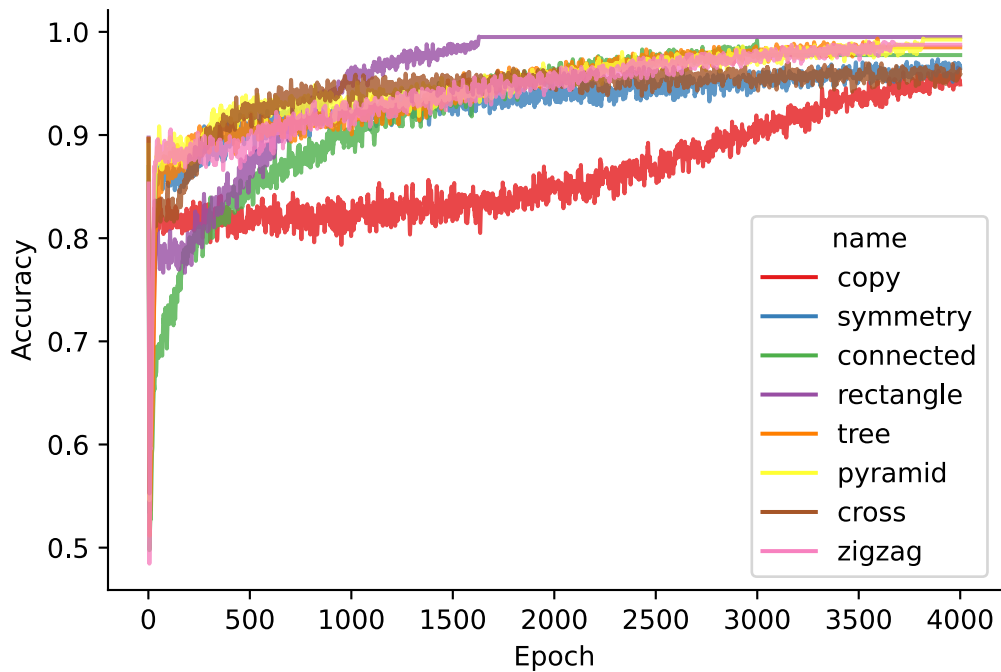

Supplement: S2 Fig — We trained one network for each abstraction. The network was trained for 4000 epochs or until the average accuracy was above 99%, whichever was first. (PDF) [file pcbi.1011316.s002.pdf]

Sweep 1

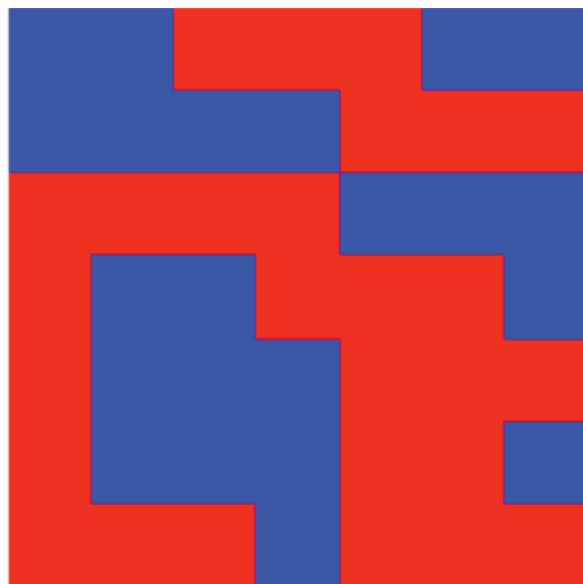

Sweep 2

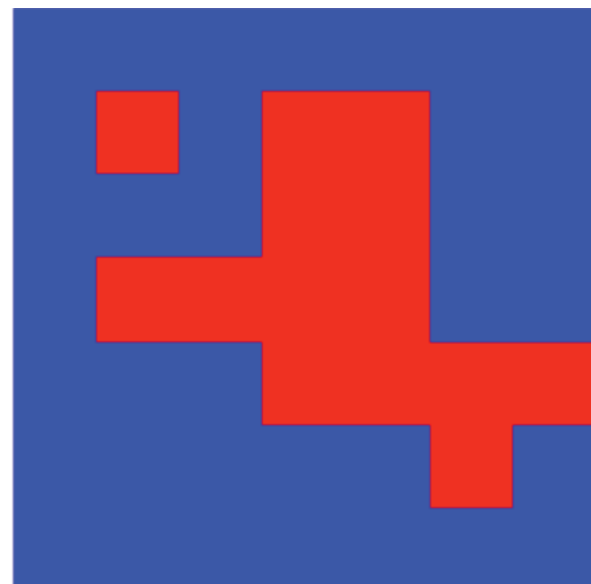

Sweep 3

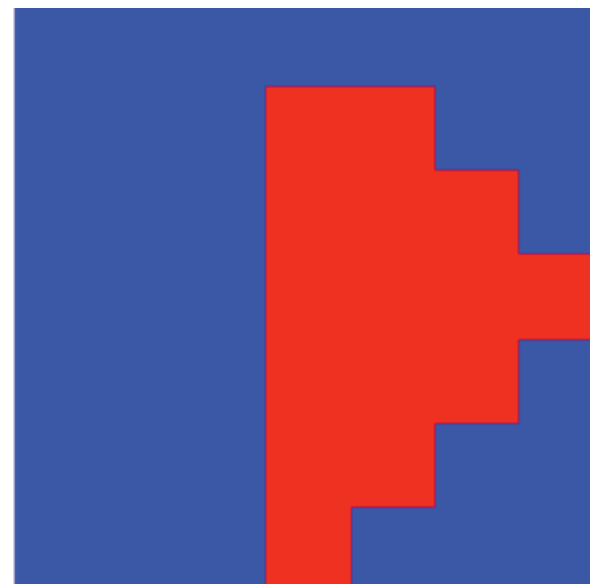

Sweep 4

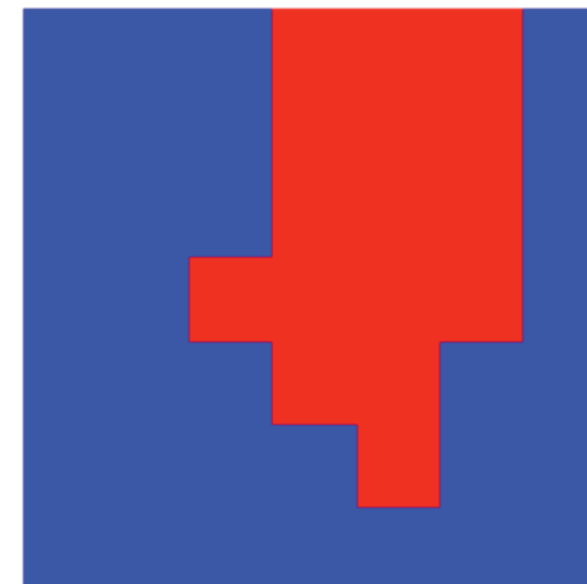

Sweep 5

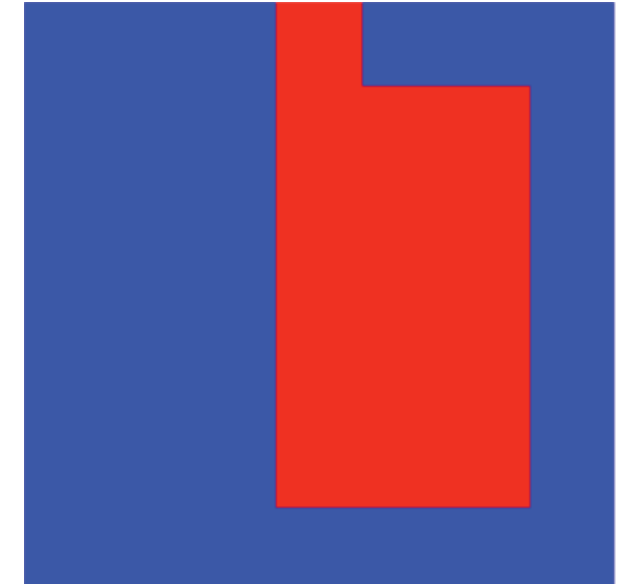

Sweep 6

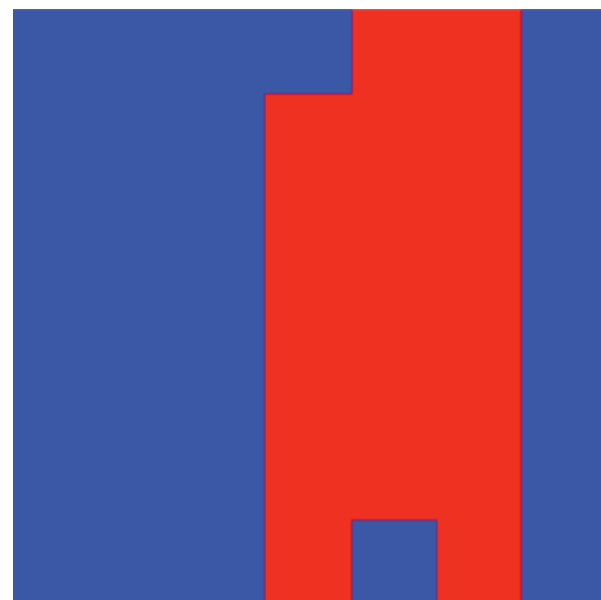

Sweep 7

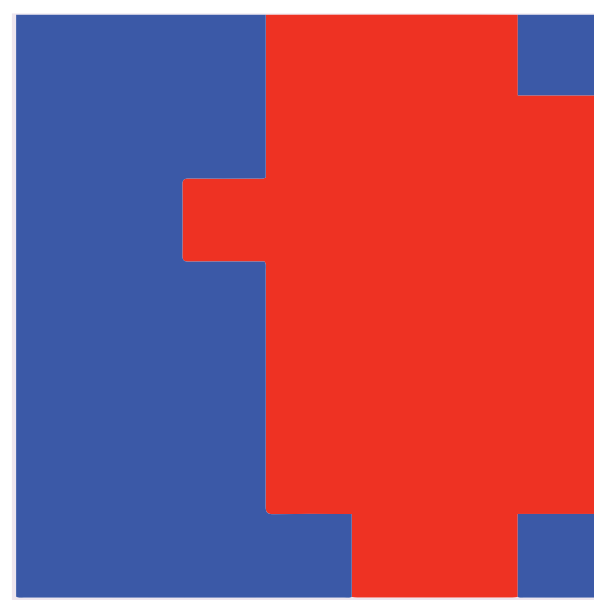

Sweep 8

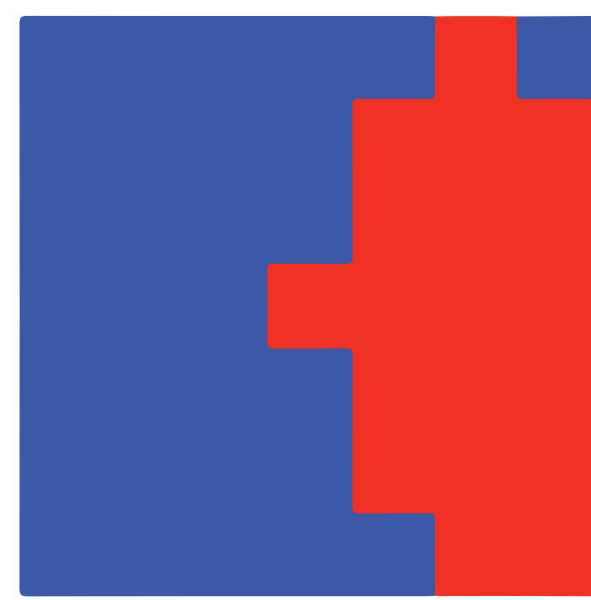

Sweep 9

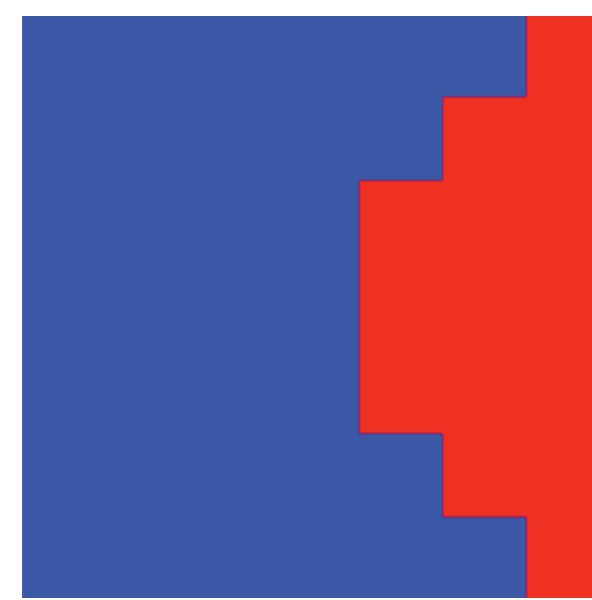

Sweep 10

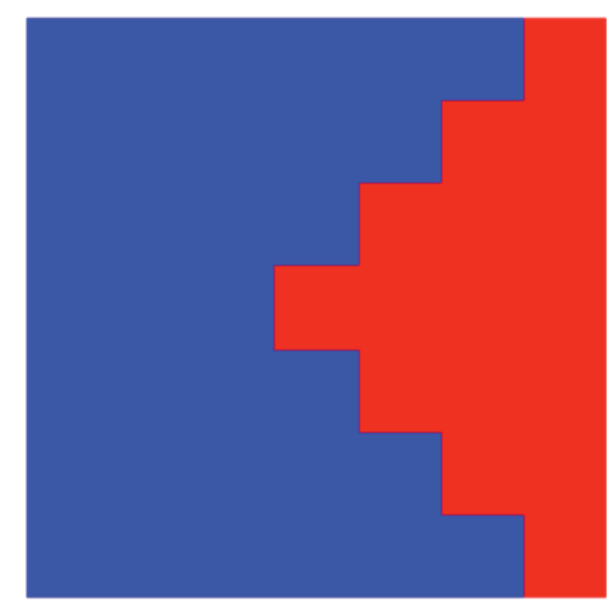

Sweep 11

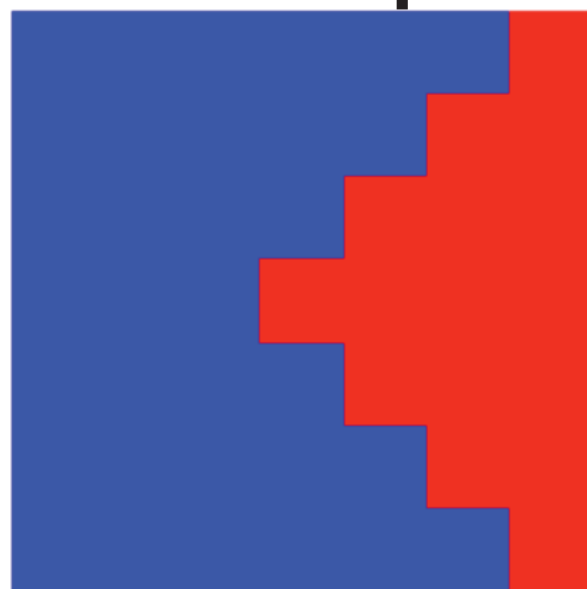

Supplement: S3 Fig — We start with a random initialization and iterate through the whole board, flipping each tile with the network’s given probability. Eleven example sweeps are shown here. (PDF) [file pcbi.1011316.s003.pdf]

## RNN Meta-Learner

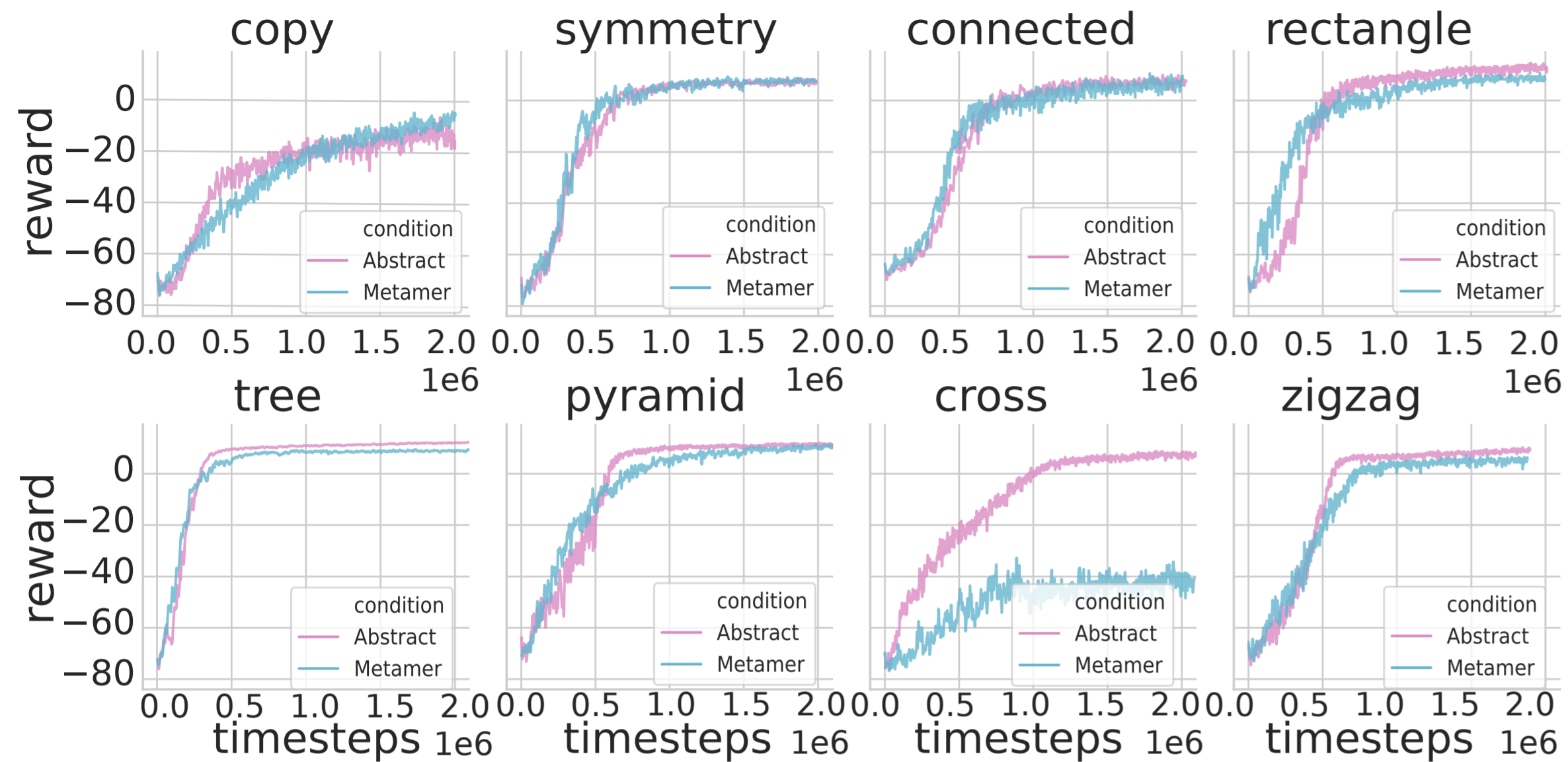

## Episodic Planning Networks

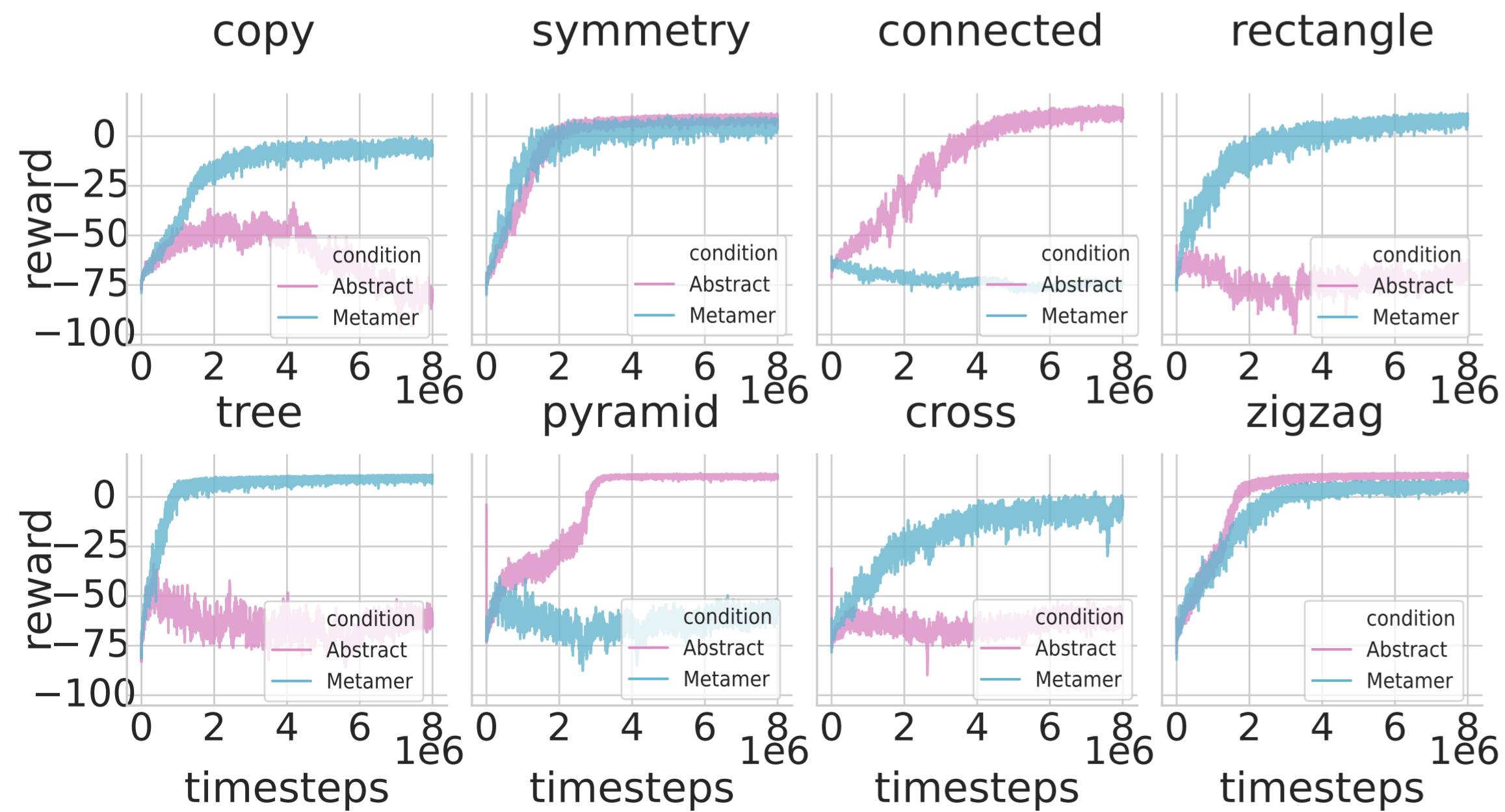

## Transformer

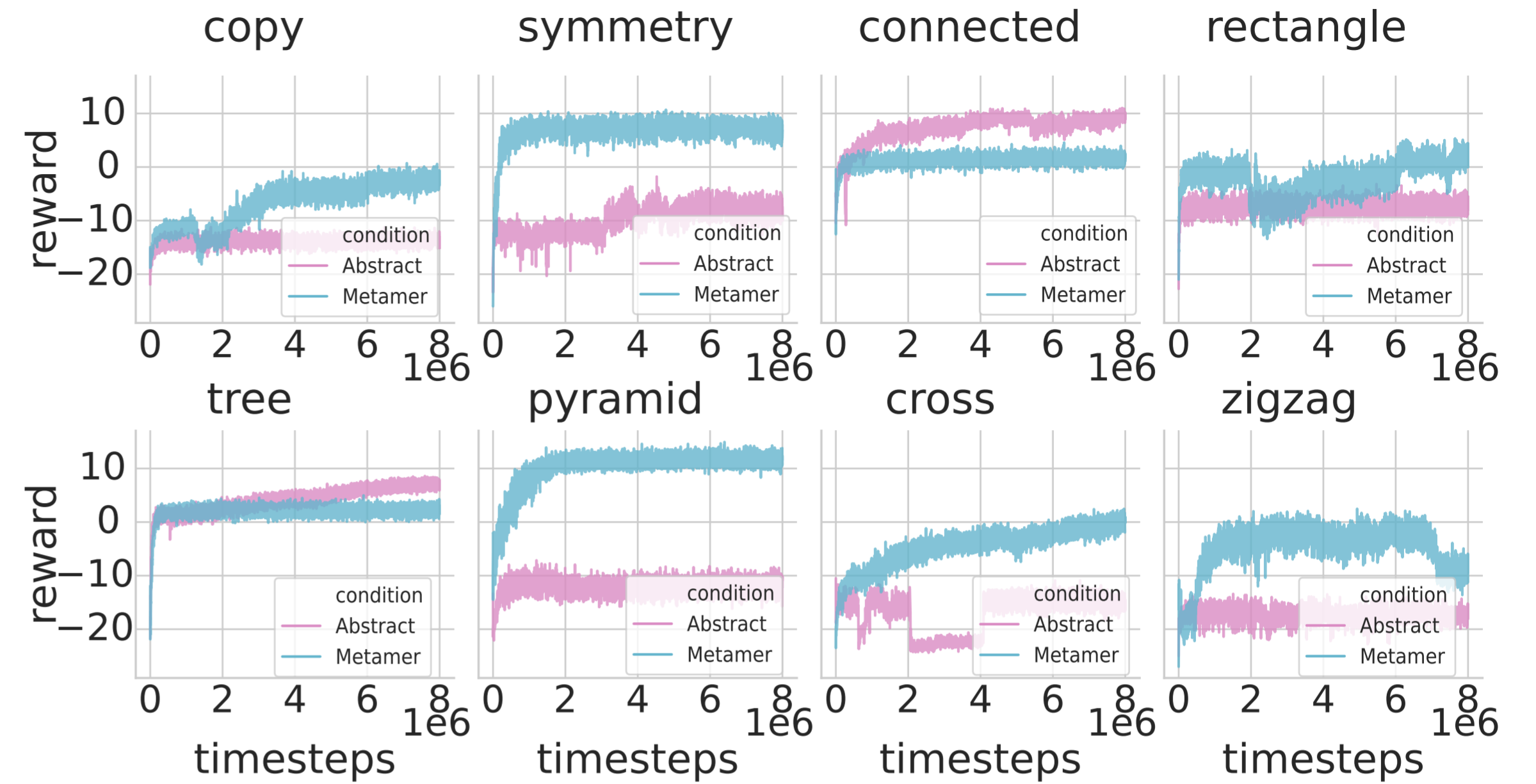

## CoReINet

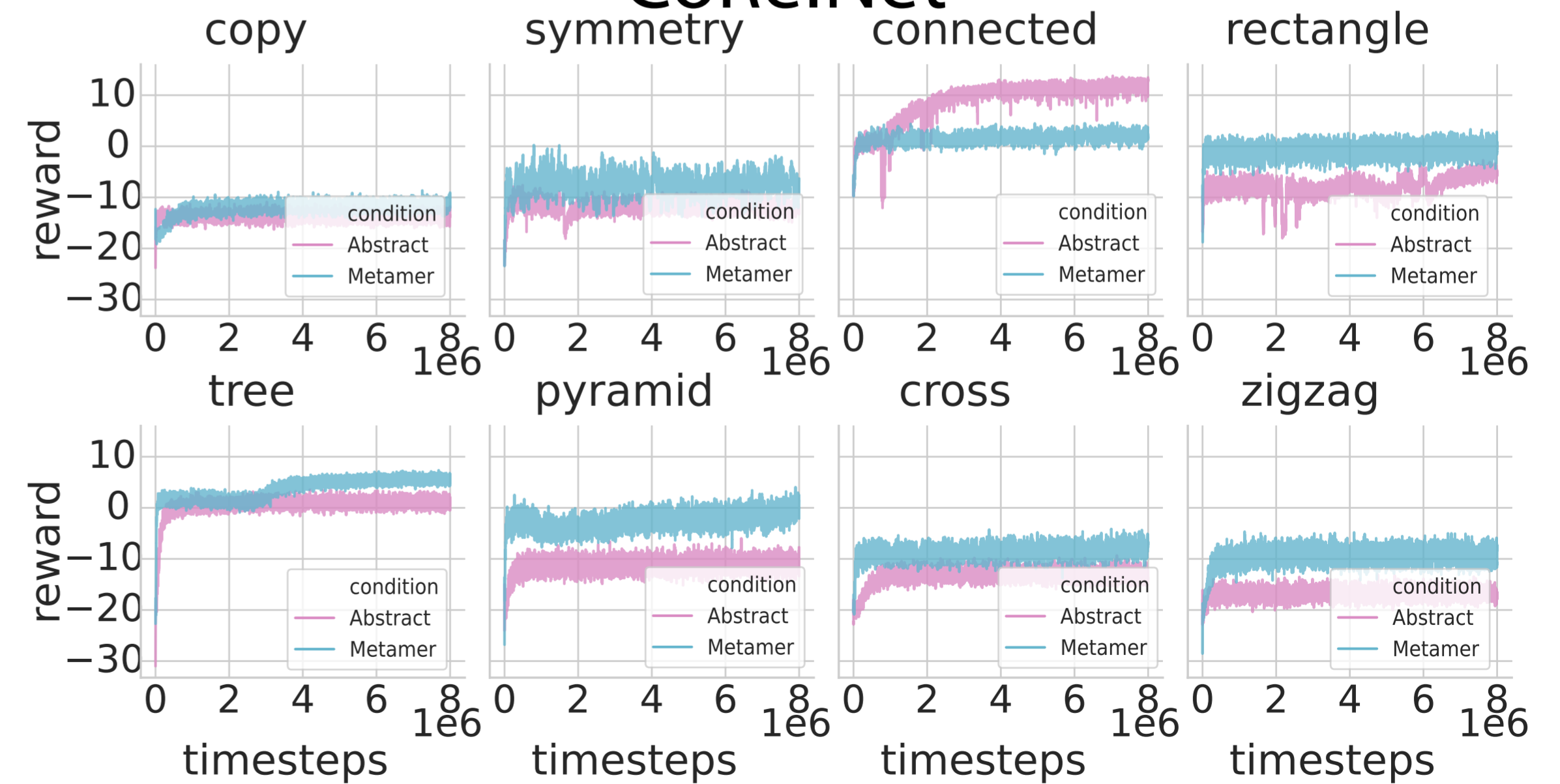

Supplement: S4 Fig — (PDF) [file pcbi.1011316.s004.pdf]

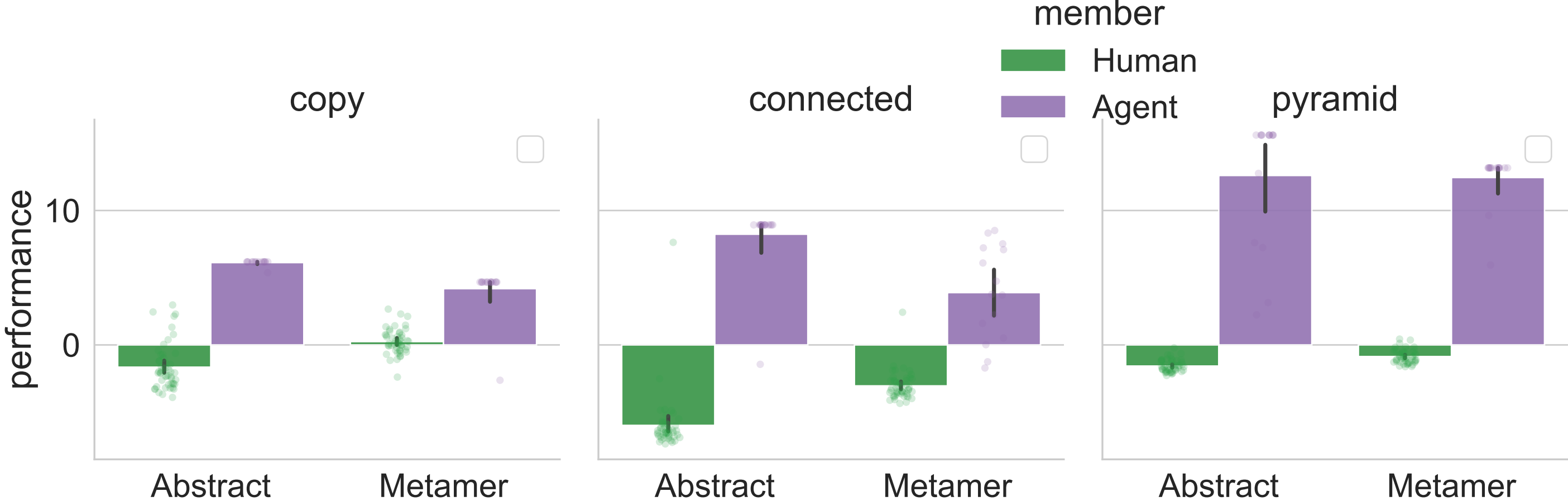

Supplement: S5 Fig — We do this on the task distributions in which we saw the largest difference between agents and humans. Note that this is an out-of-distribution test and we would normally expect that an agent would have better test performance on tasks that are from the distribution on which it was trained. We find, however, that agents performed similarly or even better on the metamer test tasks than on held-out abstract boards for some of the abstractions. This suggests that agents did not respond to the metamer boards as out of its training distribution. Rather, the agent’s behavior indicates that the metamer distribution actually shares the structure it learned during training on the abstract tasks. This is consistent with the hypothesis that the agent learns statistical features even when directly trained on abstract task distributions, learning the statistics associated with those abstractions rather than the abstractions themselves (PDF) [file pcbi.1011316.s005.pdf]

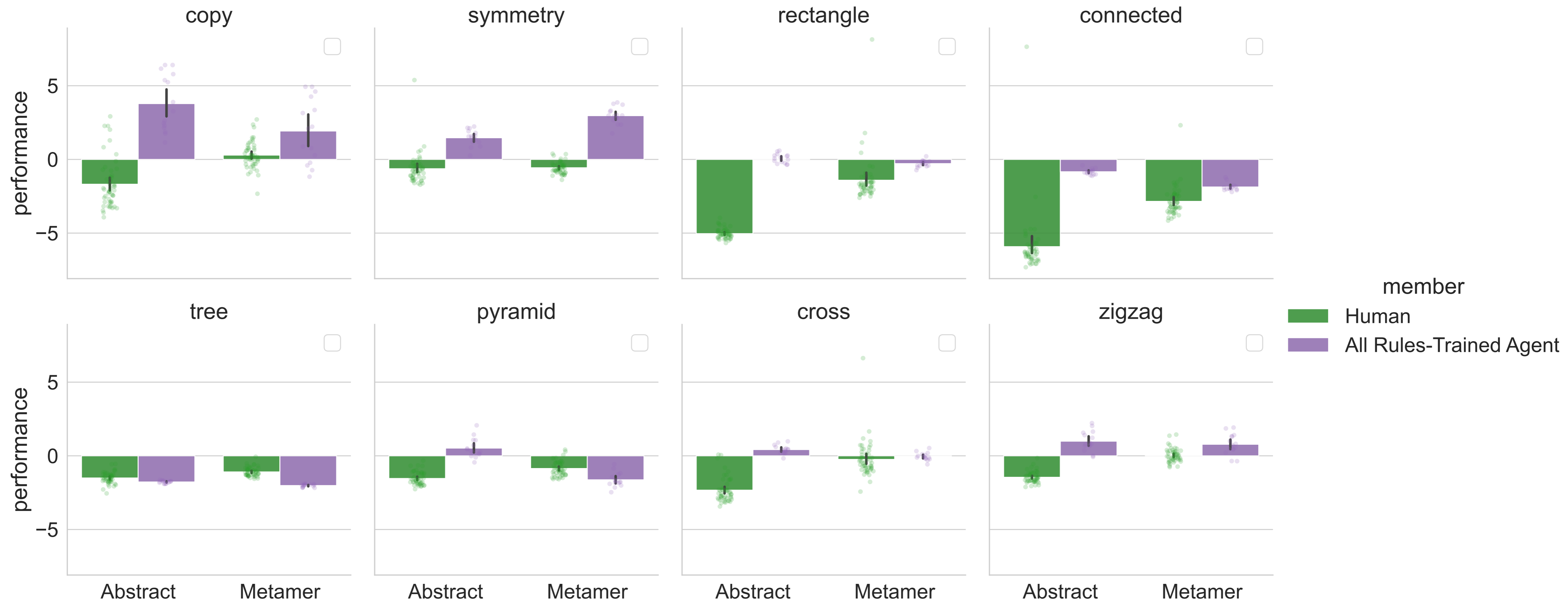

Supplement: S6 Fig — In this analysis, we trained a single agent on a mixture of all eight rules (by randomly sampling from each one of the rules during training) and evaluated it on held-out examples from each of the eight rules and their metamers. The agent consistently did the same or better on the metamer boards than the rule-based boards in seven out of the eight rules. Note that this agent did not see any metamers during training, yet often generalizes better to the metamer distribution. (PDF) [file pcbi.1011316.s006.pdf]

**A**

performance (z-scored by heuristic)

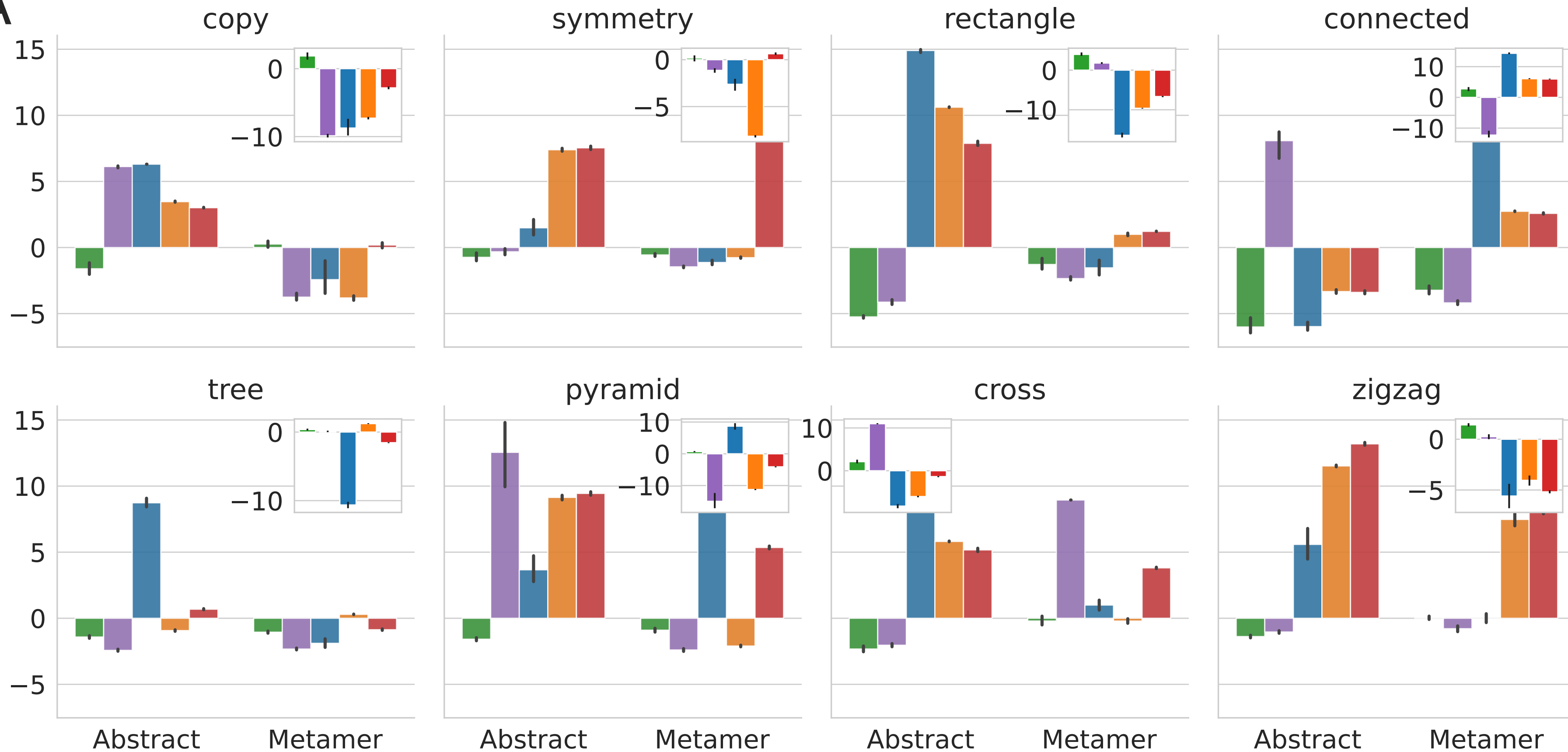**B**Metamer vs Abstract  
(Statistical Test)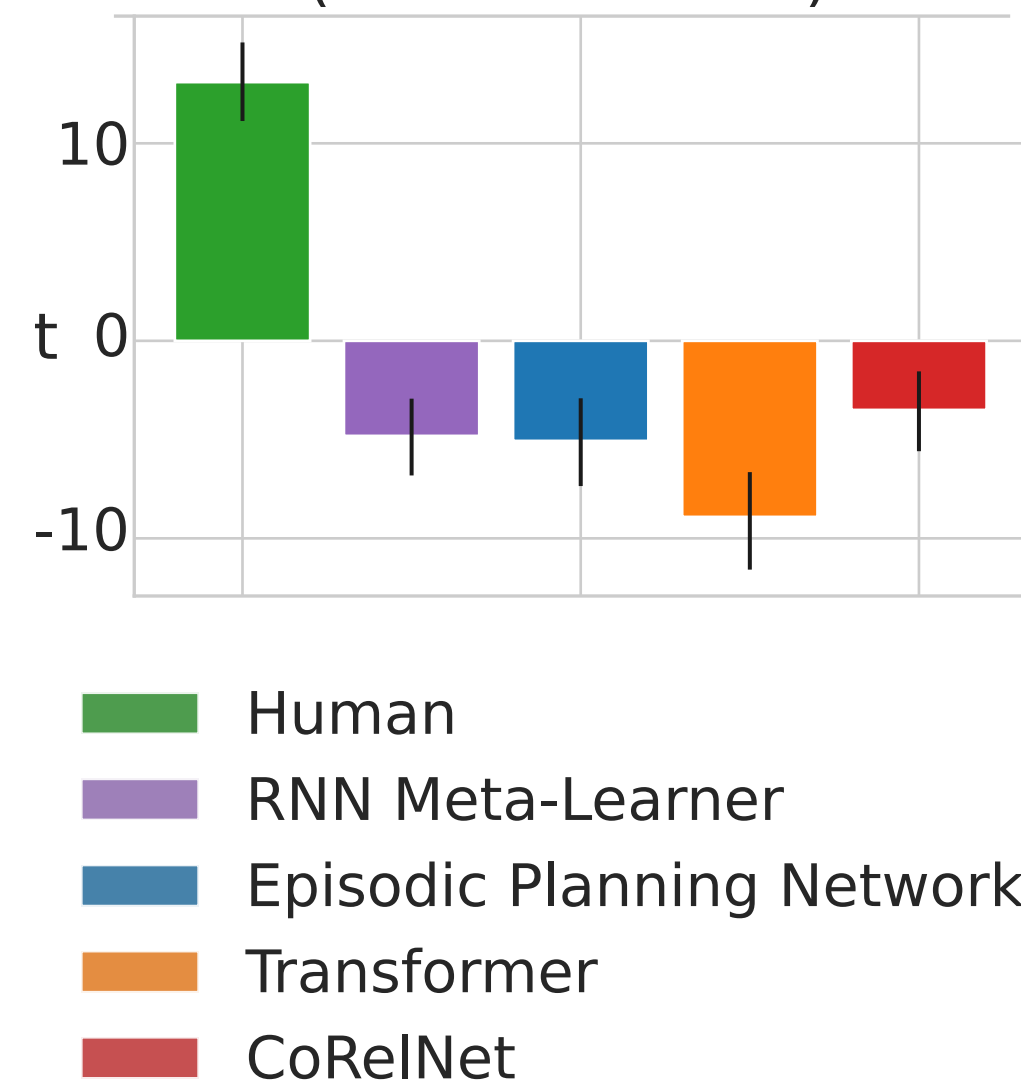

Supplement: S7 Fig — (A) Raw performance across all abstract and metamer tasks for humans and all neural network architectures. Inset plots each contain the difference between abstract and metamer performance. (B) Results from two-sample independent t-tests that compare metamer vs abstract performance on other neural network architectures. This is the same as Fig 4 reproduced here for convenience. (PDF) [file pcbi.1011316.s007.pdf]
