# Supplementary material for: Disentangling Abstraction from Statistical Pattern Matching in Human and Machine Learning
Source: PLoS Comput Biol. 2023 Aug 25;19(8):e1011316. doi: 10.1371/journal.pcbi.1011316 (PMC10497163; doi:10.1371/journal.pcbi.1011316)
Supplement: S1 Table — (PDF) [file pcbi.1011316.s009.pdf]

Abstraction	Description	Generative Process
Copy	Copy takes a random 3x3 configuration and copies it onto a random non-overlapping place on the board.	-Make a random binary array and reshape into 3x3 configuration.<br>-Insert into 7x7 grid in random location<br>-Randomly choose a 3x3 location that doesn't overlap with previous configuration and copy configuration into new location as well.
Symmetry	Symmetry contains a set of red tiles that are symmetry with respect to either the horizontal or vertical dimension.	-Choose randomly whether to do horizontal or vertical symmetry.<br>-Choose a random row or column (except the first/last row/column) to make the axis of symmetry (for horizontal and vertical respectively)<br>-Choose a random tile on the axis to color red.<br>-repeat for four times:<br>-Choose a random neighbor to a current red tile to the left/above the axis to color red.<br>-Color the reflection (across the axis) of the new red tile red as well.
Rectangle	Rectangle contains red tiles shaped as a rectangle.	-Randomly choose two points in the 7x7 grid as the upper left and rower right corners of the rectangle.<br>-Draw a rectangle using those two points (a rectangle can be completely determined given the location of two of its corners).
Connected	Connected contains a set of red tiles that are connected in one closed loop such that one can trace through the entire loop by going through each red tile exactly once (similar to a hamiltonian cycle on graphs).	-Randomly choose a tile in the board (all white at the beginning) as a start tile and color it blue.<br>-for 1-3 iterations<br>-Choose a random neighbor and label in blue.<br>This should build a random blue shape in the board. Then, color all white tiles surrounding this shape (any white tile neighboring any blue tile) red.
Tree	Trees are generated from a branching context-free grammar used in Kumar et al. 2021.	On the first tree production, the next two red tiles will either be t1 = (sx+1, sy), t2 = (sx, sy − 1) or t1 = (sx + 1, sy), t2(sx, sy − 1) or t1 = (sx − 1, sy), t2 = (sx, sy + 1) or t1 = (sx − 1, sy), t2 = (sx, sy + 1). The tree production rule always builds in two orthogonal directions. On subsequent tree productions, one of the two added red tiles from the previous production will be picked and two orthogonal directions will be picked for the next two red tiles. The defining characteristic in the tree structure is the "lack of loops", which means there can never be a 2x2 sub-square of all red tiles. Therefore, a currently red tile t is chosen for the center of production such that there exists a pair of tiles t1, t2 in orthogonal directions to t such that making both t1, t2 red does not create a 2x2 sub-square of red tiles.
Cross	Cross contains two lines (either vertical and horizontal or diagonal) that intersect at a specific tile.	-Choose randomly whether to make a diagonal or vertical/horizontal cross.<br>-Choose a random center tile and color red.

		-Choose random endpoints of the perpendicular segments that meet at the center tile and color red.
Pyramid	Pyramid contains red tiles that are in the shape of a pyramid, where there is a base of red tiles on the bottom of a triangular structure converging to a one tile row on the top. Pyramids can sometimes be rotated 90 degrees.	-Select a base size of either size 7, 5, or 3.<br>-Select a random row to be the base location. If its size 7, it must be at the bottom. If it is size 5, it must have at least 3 rows above.<br>-Build up the rest of the pyramid (either has 4 levels if base size is 7, 3 if base size is 5, or 2 if base size is 1).<br>-Randomly apply a rotation (0/90/180/270 degrees).
Zigzag	Zigzag contains red tiles in the shape of a zigzag, which is a series of connected straight lines in alternating directions.	-Choose a random start location<br>-Choose a step size between 1,2,3,4,5, or 6 (the max possible step size depends on the start location).<br>-while you haven't reached the end of the board:<br>-Color all tiles *step* tiles away horizontally red.<br>-Color all tiles *step* tiles away vertically red to complete one zigzag.<br>-shift the "tile of focus" as the tile at the end of zigzag (so [x,y]->[x+step,y+step]
