# Supplementary material for: Disentangling Abstraction from Statistical Pattern Matching in Human and Machine Learning
Source: PLoS Comput Biol. 2023 Aug 25;19(8):e1011316. doi: 10.1371/journal.pcbi.1011316 (PMC10497163; doi:10.1371/journal.pcbi.1011316)
Supplement: S2 Table — (PDF) [file pcbi.1011316.s010.pdf]

Factor	df	F-value	P-Value
Human vs Agent	1	734.751802	<0.001
Abstraction	7	174.756578	<0.001
Abstract vs Metamer	1	66.609924	<0.001
Human vs Agent / Abstract vs Metamer	1	903.012393	<0.001
Human vs Agent / Abstract Rule	7	183.163312	<0.001
Abstract Rule / Abstract vs Metamer	7	140.821103	<0.001
Human vs Agent / Abstract vs Metamer / Abstract Rule	7	335.446094	<0.001
