# Supplementary material for: Disentangling Abstraction from Statistical Pattern Matching in Human and Machine Learning
Source: PLoS Comput Biol. 2023 Aug 25;19(8):e1011316. doi: 10.1371/journal.pcbi.1011316 (PMC10497163; doi:10.1371/journal.pcbi.1011316)
Supplement: S3 Table — (PDF) [file pcbi.1011316.s011.pdf]

Abstraction	df	F-value	P-value
Copy	1	562.395343	<0.001
Symmetry	1	20.815571	<0.001
Connected	1	478.523676	<0.001
Rectangle	1	18.925626	<0.001
Zigzag	1	49.480372	<0.001
Tree	1	11.052533	0.0032
Pyramid	1	483.322974	<0.001
Cross	1	465.263164	<0.001
