# Supplementary material for: Disentangling Abstraction from Statistical Pattern Matching in Human and Machine Learning
Source: PLoS Comput Biol. 2023 Aug 25;19(8):e1011316. doi: 10.1371/journal.pcbi.1011316 (PMC10497163; doi:10.1371/journal.pcbi.1011316)
Supplement: S4 Table — (PDF) [file pcbi.1011316.s012.pdf]

Task Distribution	Human Abstract vs Human Metamer (t)	Human Abstract vs Human Metamer (p)
copy	-7.05802	<0.001
symmetry	-0.93046	0.35442
rectangle	-16.77618	<0.001
connected	-9.1196	<0.001
tree	-6.31362	<0.001
pyramid	-7.01523	<0.001
cross	-10.1344	<0.001
zigzag	-16.89938	<0.001

Task Distribution	Agent Metamer vs Agent Abstract (t)	Agent Metamer vs Agent Abstract (p)
copy	-68.47882	<0.001
symmetry	-9.38291	<0.001
rectangle	15.07433	<0.001
connected	-18.08568	<0.001
tree	0.82062	0.41879
pyramid	-11.74115	<0.001
cross	164.87218	<0.001
zigzag	1.47712	0.1508

Task Distribution	Human Abstract vs Agent Abstract (t)	Human Abstract vs Agent Abstract (p)
copy	-17.84887	<0.001
symmetry	-1.17116	0.24595
rectangle	-10.40058	<0.001
connected	-21.52446	<0.001
tree	9.09121	<0.001
pyramid	-20.49073	<0.001
cross	-1.47796	0.1444
zigzag	-4.23903	<0.001

Task Distribution	Agent Metamer vs Human Metamer (t)	Agent Metamer vs Human Metamer (p)
copy	-16.26451	<0.001
symmetry	-9.23867	<0.001

rectangle	-2.4756	0.016
connected	-4.92417	<0.001
tree	-15.91022	<0.001
pyramid	-10.42991	<0.001
cross	27.63812	<0.001
zigzag	-6.19551	<0.001
