# Supplementary material for: Disentangling Abstraction from Statistical Pattern Matching in Human and Machine Learning
Source: PLoS Comput Biol. 2023 Aug 25;19(8):e1011316. doi: 10.1371/journal.pcbi.1011316 (PMC10497163; doi:10.1371/journal.pcbi.1011316)
Supplement: S5 Table — (PDF) [file pcbi.1011316.s013.pdf]

Architecture	Factor	df	F-value	P-Value
EPN	Human vs Agent	1	4795.51026	<0.001
EPN	Abstraction	7	184.991208	<0.001
EPN	Abstract vs Metamer	1	45.5277506	<0.001
EPN	Human vs Agent / Abstract vs Metamer	1	1176.12352	<0.001
EPN	Human vs Agent / Abstract Rule	7	206.512069	<0.001
EPN	Abstract Rule / Abstract vs Metamer	7	169.026822	<0.001
EPN	Human vs Agent / Abstract vs Metamer / Abstract Rule	7	582.310177	<0.001
Transformer	Human vs Agent	1	4965.84711	<0.001
Transformer	Abstraction	7	354.320165	<0.001
Transformer	Abstract vs Metamer	1	7.70838643	0.00556
Transformer	Human vs Agent / Abstract vs Metamer	1	2388.50291	<0.001
Transformer	Human vs Agent / Abstract Rule	7	325.948355	<0.001
Transformer	Abstract Rule / Abstract vs Metamer	7	116.322346	<0.001
Transformer	Human vs Agent / Abstract vs Metamer / Abstract Rule	7	242.213245	<0.001
CoRelNet	Human vs Agent	1	8699.14334	<0.001
CoRelNet	Abstraction	7	495.918709	<0.001
CoRelNet	Abstract vs Metamer	1	229.943473	<0.001
CoRelNet	Human vs Agent / Abstract vs Metamer	1	712.617936	<0.001
CoRelNet	Human vs Agent / Abstract Rule	7	342.041602	<0.001

CoRelNet	Abstract Rule / Abstract vs Metamer	7	65.1817456	<0.001
CoRelNet	Human vs Agent / Abstract vs Metamer / Abstract Rule	7	127.423545	<0.001
