# Supplementary material for: Disentangling Abstraction from Statistical Pattern Matching in Human and Machine Learning
Source: PLoS Comput Biol. 2023 Aug 25;19(8):e1011316. doi: 10.1371/journal.pcbi.1011316 (PMC10497163; doi:10.1371/journal.pcbi.1011316)
Supplement: S6 Table — (PDF) [file pcbi.1011316.s014.pdf]

Architecture	Abstraction	F-value	P-value
EPN	copy	307.545314	<0.001
EPN	symmetry	64.271204	<0.001
EPN	connected	341.487512	<0.001
EPN	rectangle	2035.410532	<0.001
EPN	zigzag	348.068599	<0.001
EPN	tree	3736.383497	<0.001
EPN	pyramid	586.232387	<0.001
EPN	cross	609.208821	<0.001
Transformer	copy	340.637725	<0.001
Transformer	symmetry	760.781090	<0.001
Transformer	connected	28.090256	<0.001
Transformer	rectangle	1045.388918	<0.001
Transformer	zigzag	673.054264	<0.001
Transformer	tree	48.462158	<0.001
Transformer	pyramid	4368.560408	<0.001
Transformer	cross	415.253415	<0.001
CoRelNet	copy	89.408661	<0.001
CoRelNet	symmetry	2.243686	0.136662
CoRelNet	connected	26.478893	<0.001
CoRelNet	rectangle	634.874681	<0.001
CoRelNet	zigzag	1666.695083	<0.001
CoRelNet	tree	237.142038	<0.001
CoRelNet	pyramid	713.356633	<0.001
CoRelNet	cross	76.242829	<0.001
