# Supplementary material for: Disentangling Abstraction from Statistical Pattern Matching in Human and Machine Learning
Source: PLoS Comput Biol. 2023 Aug 25;19(8):e1011316. doi: 10.1371/journal.pcbi.1011316 (PMC10497163; doi:10.1371/journal.pcbi.1011316)
Supplement: S7 Table — (PDF) [file pcbi.1011316.s015.pdf]

Architecture	Rule	Agent Metamer vs Agent Abstract (t)	Agent Metamer vs Agent Abstract (p)
EPN	copy	-13.488338	<0.001
EPN	symmetry	-7.9001985	<0.001
EPN	connected	93.9352362	<0.001
EPN	rectangle	-52.017198	<0.001
EPN	zigzag	-8.7948368	<0.001
EPN	tree	-43.433857	<0.001
EPN	pyramid	16.5289277	<0.001
EPN	cross	-31.678034	<0.001
Transformer	copy	-76.293361	<0.001
Transformer	symmetry	-120.73577	<0.001
Transformer	connected	80.3191281	<0.001
Transformer	rectangle	-183.1902	<0.001
Transformer	zigzag	-15.345424	<0.001
Transformer	tree	36.9415572	<0.001
Transformer	pyramid	-111.03095	<0.001
Transformer	cross	-64.277525	<0.001
CoRelNet	copy	-26.637629	<0.001
CoRelNet	symmetry	8.62732174	<0.001
CoRelNet	connected	88.5209542	<0.001
CoRelNet	rectangle	-77.693863	<0.001
CoRelNet	zigzag	-64.549005	<0.001
CoRelNet	tree	-40.919702	<0.001
CoRelNet	pyramid	-45.481782	<0.001
CoRelNet	cross	-17.749355	<0.001
