# Supplementary material for: Disentangling Abstraction from Statistical Pattern Matching in Human and Machine Learning
Source: PLoS Comput Biol. 2023 Aug 25;19(8):e1011316. doi: 10.1371/journal.pcbi.1011316 (PMC10497163; doi:10.1371/journal.pcbi.1011316)
Supplement: S8 Table — (PDF) [file pcbi.1011316.s016.pdf]

model	task	gamma	n_steps	Lr schedue	lr	ent_coef	vf_coef	attndim	numhead	memsize	num layer	dropout
RNN Meta-Learner	copy	0.9	2	constant	0.00062239	0.00243152	0.0010111					
RNN Meta-Learner	copy_null	0.9	2	constant	0.0003057	0.00851215	0.01116498					
RNN Meta-Learner	symmetry	0.9	4	constant	0.00131308	0.00513281	0.01843889					
RNN Meta-Learner	symmetry_null	0.9	4	linear	0.00117687	0.00197973	0.01707586					
RNN Meta-Learner	connected	0.9	6	constant	0.00209519	0.0618838	0.01825565					
RNN Meta-Learner	connected_null	0.9	2	linear	0.00083198	0.00963399	0.02033728					
RNN Meta-Learner	rectangle	0.9	2	constant	0.00123548	1.20E-08	0.02693679					
RNN Meta-Learner	rectangle_null	0.9	4	linear	0.00108637	0.01587278	0.02786049					
RNN Meta-Learner	tree	0.9	10	linear	0.00377961	0.04569161	0.01020331					
RNN Meta-Learner	tree_null	0.9	10	constant	0.00142225	0.00014317	0.01032152					
RNN Meta-Learner	pyramid	0.9	2	constant	0.00094057	0.00077839	0.01478618					
RNN Meta-Learner	pyramid_null	0.9	6	linear	0.00093163	0.00065686	0.02547757					
RNN Meta-Learner	cross	0.9	2	linear	0.00045011	0.00059439	0.00407202					
RNN Meta-Learner	cross_null	0.9	2	linear	0.00080997	3.88E-06	0.06421612					
RNN Meta-Learner	zigzag	0.9	4	constant	0.00165426	4.21E-07	0.03229156					

RNN Meta-Learner	zigzag_null	0.9	4	constant	0.00075596	0.00061885	0.03651008					
Episodic Planning Networks	copy_null	0.995	2	constant	0.00033313	6.54E-06	0.77188886	16	2	10		
Episodic Planning Networks	copy_null	0.9	2	constant	0.00014579	0.00225419	0.36511053	16	1	10		
Episodic Planning Networks	symmetry_null	0.9	2	constant	8.16E-05	4.16E-05	0.40127644	64	4	20		
Episodic Planning Networks	symmetry_null	0.9	2	constant	0.00022314	0.00053168	0.57356288	32	1	20		
Episodic Planning Networks	connected_null	0.98	2	linear	0.00038639	0.01586469	0.37999232	16	2	5		
Episodic Planning Networks	connected_null	0.95	2	linear	0.00064304	0.01233968	0.74103176	32	8	40		
Episodic Planning Networks	rectangle_null	0.98	2	linear	0.00058851	7.44E-08	0.54919912	16	2	10		
Episodic Planning Networks	rectangle_null	0.95	4	constant	0.00032927	0.00148335	0.33773876	16	4	20		
Episodic Planning Networks	tree_null	0.9	2	linear	0.00044907	4.95E-08	0.66245802	32	4	40		
Episodic Planning Networks	tree_null	0.9	6	linear	0.00077876	1.19E-06	0.19450061	16	1	20		
Episodic Planning Networks	pyramid_null	0.95	2	constant	0.00017242	0.09845655	0.87941841	16	2	5		
Episodic Planning Networks	pyramid_null	0.9	2	linear	0.00047711	1.26E-05	0.85291822	32	1	10		
Episodic Planning Networks	cross_null	0.99	2	linear	0.00041926	0.00879789	0.72213536	32	8	10		
Episodic Planning Networks	cross_null	0.98	2	constant	0.00027736	0.00012245	0.11840353	16	4	20		
Episodic Planning Networks	zigzag_null	0.9	2	constant	0.00010779	9.44E-06	0.25689978	16	4	20		

Episodic Planning Networks	zigzag_null	0.95	2	constant	8.47E-05	0.00193906	0.12524522	64	2	20		
Transformers	copy_null	0.995	10	constant	6.11E-05	0.00013691	0.43870801	16	2		9	0.15
Transformers	copy_null	0.95	14	constant	0.00011745	0.00013081	0.32088709	64	1		9	0.15
Transformers	symmetry_null	0.95	2	linear	0.00015481	2.13E-05	0.06865333	64	2		7	0.15
Transformers	symmetry_null	0.98	2	linear	0.0007978	0.0180259	0.20609321	16	1		9	0
Transformers	connected_null	0.9	14	constant	0.00037219	1.43E-06	0.27743182	32	8		3	0.15
Transformers	connected_null	0.999	6	constant	4.41E-05	9.80E-05	0.43860151	16	2		7	0.15
Transformers	rectangle_null	0.995	4	linear	0.00015984	0.00013658	0.62287718	64	1		3	0.15
Transformers	rectangle_null	0.98	6	constant	0.00013261	0.00031288	0.13047689	16	2		9	0.15
Transformers	tree_null	0.99	8	constant	0.00011141	8.68E-08	0.81537314	32	4		3	0.15
Transformers	tree_null	0.999	6	constant	4.41E-05	9.80E-05	0.43860151	16	2		7	0.15
Transformers	pyramid_null	0.95	8	linear	2.84E-05	0.00332861	0.37986521	16	1		2	0.15
Transformers	pyramid_null	0.9	2	constant	0.00055707	0.01880732	0.26666269	16	8		3	0
Transformers	cross_null	0.95	6	linear	0.00095565	1.03E-07	0.48805628	32	2		3	0.15
Transformers	cross_null	0.95	14	constant	4.90E-05	0.09286513	0.05653123	64	1		9	0.15
Transformers	zigzag_null	0.99	2	linear	8.40E-05	0.00132145	0.03231586	64	2		9	0.15
Transformers	zigzag_null	0.995	2	linear	0.00062963	0.0121549	0.67292639	16	2		7	0

CoRelNet	copy_null	0.995	2	linear	0.00017245	3.01E-06	0.91449804					
CoRelNet	copy_null	0.95	6	linear	1.32E-05	0.00541178	0.68377464					
CoRelNet	symmetry_null	0.999	14	linear	0.00085595	0.08905802	0.52761016					
CoRelNet	symmetry_null	0.9999	8	linear	0.00070923	1.74E-06	0.50732897					
CoRelNet	connected_null	0.9	10	constant	0.00094003	4.76E-08	0.67602848					
CoRelNet	connected_null	0.99	14	linear	0.00037109	0.00624795	0.22856862					
CoRelNet	rectangle_null	0.99	6	linear	0.00080133	1.06E-08	0.41778158					
CoRelNet	rectangle_null	0.999	6	linear	0.00148068	5.47E-05	0.41924005					
CoRelNet	tree_null	0.95	4	linear	2.27E-05	0.00673502	0.77815675					
CoRelNet	tree_null	0.999	6	linear	0.00024816	0.0368649	0.01963117					
CoRelNet	pyramid_null	0.995	4	linear	3.88E-05	1.91E-08	0.84543826					
CoRelNet	pyramid_null	0.95	4	constant	0.00015848	1.20E-05	0.5270834					
CoRelNet	cross_null	0.9	12	linear	2.57E-05	1.95E-08	0.71630464					
CoRelNet	cross_null	0.95	10	constant	0.00019137	7.39E-05	0.51232182					
CoRelNet	zigzag_null	0.95	8	linear	0.00011151	1.07E-05	0.28885087					
CoRelNet	zigzag_null	0.999	4	linear	2.76E-05	0.00033608	0.246342					
