# Supplementary material for: Disentangling Abstraction from Statistical Pattern Matching in Human and Machine Learning
Source: PLoS Comput Biol. 2023 Aug 25;19(8):e1011316. doi: 10.1371/journal.pcbi.1011316 (PMC10497163; doi:10.1371/journal.pcbi.1011316)
Supplement: S8 Fig — Each cell denotes the z-score performance (with respect to the nearest neighbor heuristic, as in Fig 3A) of the agent when trained on the respective rule of the row and tested on the respective rule on the column. One interesting finding from this map is that all of the training rules produce comparable performance on the copy task, which may suggest the difficulty in learning the unique structure that the copy task employs. Another interesting finding is that training on zigzag metamers leads to good performance on all other metamer tasks, suggesting that the statistics within the zigzag metamers helps the network learn all the other metamer tasks. (PDF) [file pcbi.1011316.s008.pdf]

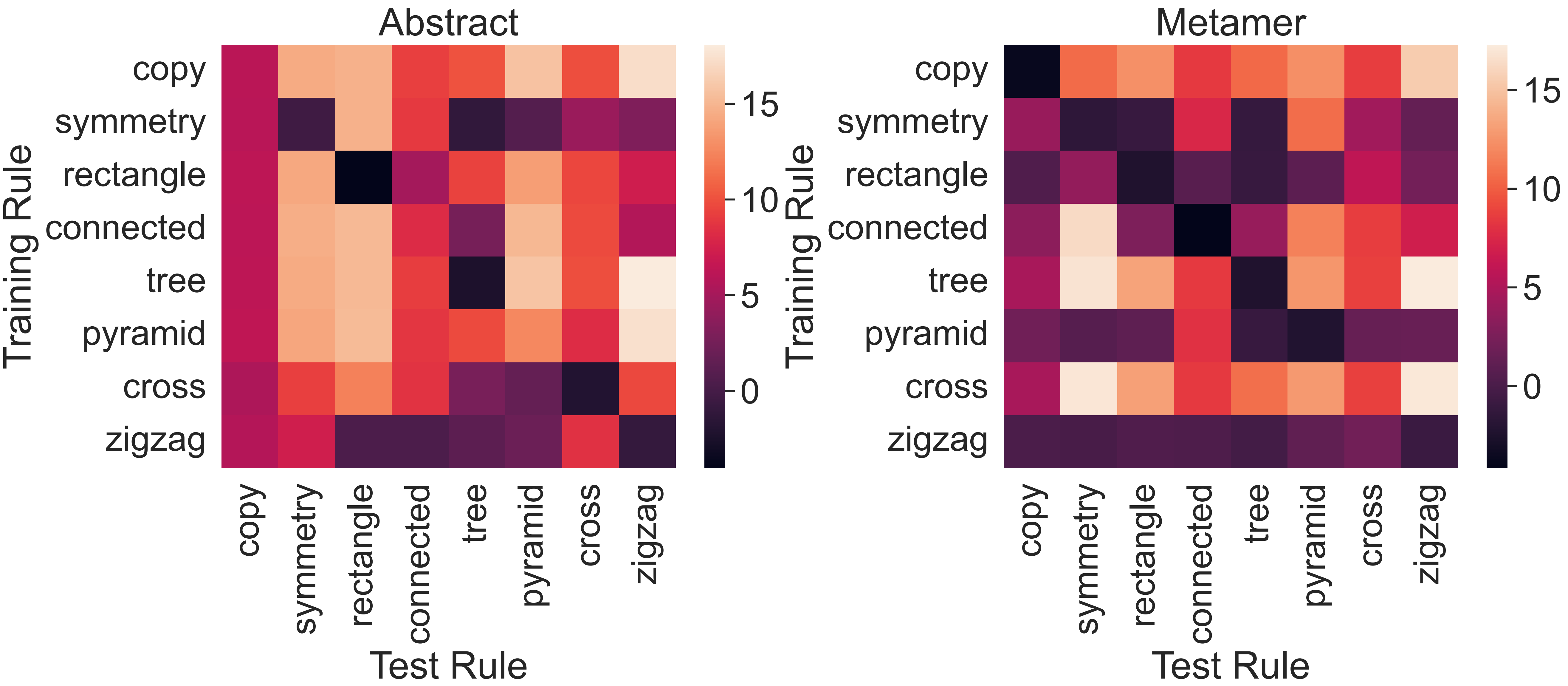
Abstract
Metamer
Training Rule
Test Rule
copy
symmetry
rectangle
connected
tree
pyramid
cross
zigzag
15
10
5
0
