# Supplementary material for: Disentangling Abstraction from Statistical Pattern Matching in Human and Machine Learning
Source: PLoS Comput Biol. 2023 Aug 25;19(8):e1011316. doi: 10.1371/journal.pcbi.1011316 (PMC10497163; doi:10.1371/journal.pcbi.1011316)
Supplement: Attachment — Submitted filename: Metamers_Revision_Response.pdf [file pcbi.1011316.s017.pdf]

Dear Drs. Stefano Palminteri and Daniele Marinazzo,

Thank you for organizing the review of our manuscript “Disentangling Abstraction from Statistical Pattern Matching in Human and Machine Learning” and for inviting us to submit a revision. Below we provide point-by-point responses to all of the reviewer comments (our reviewer comments in black text, our responses in red text) and highlight where in the manuscript changes were made. We have added new analyses and supplementary figures which greatly strengthen our results as well as additional text in the manuscript clarifying important points that came up in the reviews. We are grateful for the highly constructive suggestions from the reviewers, which we believe considerably improved the manuscript. We hope these changes will bring our manuscript closer to publication.

Sincerely,

Sreejan Kumar (on behalf of the authors)

==

Reviewer #1: Review of Disentangling Abstraction from Statistical Pattern Matching in Human and Machine Learning by Kumar et al

This is a clever study which explores how humans and neural networks learn to perform an RL task reminiscent of Battleships. The task involves predicting the location of hidden tiles on a grid; the tiles obey canonical (researcher-generated) placement rules, for example that they form continuous contour that traces the edge of rectangle, or they cluster together in islands that are symmetrical. Whilst these researcher-generated rules have human-recognisable rule-based semantics, tile configurations that obey each of these rules can also be considered to be samples from a generative model that builds tile grids based on patterns of 1st, 2nd and 3rd order spatial co-occurrence (although the true rules are candidate samples from this model, not all samples from this model will precisely obey the researcher-defined rules). A generative model that encodes these patterns of co-occurrence can thus be used to generate task “metamers”, allowing the researchers to whether neural networks (for example, meta-trained deep networks attempting to find the battleship in a minimal number of moves) really encode the rule-based semantics or whether they rely on the spatial statistics. As the authors put it: “We hypothesize that even on distributions of tasks generated from abstract rules, neural-network meta-learners do not necessarily internalize the abstract structure of those rules, despite performing well. Rather, they may learn the (potentially more complex) statistical structure of the stimuli and corresponding responses that are associated with the rules, without encoding the abstract rules themselves. This is in contrast with humans, who are posited (innately and/or through lifelong learning) to represent, identify, and use abstract rules in such settings (Lake et al. 2015; Lake & Piantadosi 2020; Johnson et al. 2021)”

The authors tackle this by training an additional neural network to behave as the encoder and then sampling successively from this network to produce task metamers, i.e. boards for which the conditional probability of each tile matched that predicted by the encoder. They then verified that there was indeed a good match up to the 3rd order. They then tested humans and (individually) trained meta-RL agents on both rule-based (“abstract”) and metamer boards, showing that in most cases, humans do fairly well on both, whereas the networks make significant numbers of errors on the “abstract” stimuli, which the authors interpret as implying that the networks have encoded the statistics but not the rules.

I think this study will be widely read and cited because it very elegantly makes a simple (and perhaps under-appreciated) point: that where stimuli are generated by hard-and-fast rules,

learning the feature statistics can give the misleading impression that the task has been mastered. This is just as true in object recognition (e.g. Jagadeesh 2020) as it is in the current task. Of course, given that the dependencies in the rule-based tasks are special cases of those sampled from the conditional distribution of spatial statistics, the fact you can't go backwards from the latter to the former is perhaps unsurprising in hindsight. But I think the study is valuable, nonetheless.

I did have few questions about the data that other readers might share, and a few suggestions that go beyond the current report (which I thought interesting but are not essential for the paper)

1/ performance data are normalised using a control model that randomly samples nearby tiles. I wondered if this partly distorted the pattern of naïve rewards obtained in each condition. Surely, if the spatial statistics of the metamers and rule-based tasks are strictly matched, and the claim is that the networks are simply learning the conditional distribution (and not the abstract rules), then they should do equally well on metamers and abstract tasks (whereas humans should do better on the latter because they share priors with the authors, who made the tasks?). But in fig 3A we see that networks do vastly worse on rule-based tasks. Or is this because it is simply harder to generalise these tasks because the training distribution is more narrow?

We thank the reviewer for their positive comments on our manuscript. We would like to note that the sense in which our control tasks are metamers is that even though they are similar according to some simple statistical measures (e.g. the statistics we plot in Fig. 2C), they elicit very different behaviors between systems with different inductive biases (e.g. humans vs. artificial agents). Regarding the fact that the agents do better at metamer tasks, this could be for a few different reasons. Perhaps the rules within the abstract tasks are only easily learnable with certain prior knowledge (e.g. the concept of what a rectangle is) which, as the reviewer suggests, cannot easily be learnt from the data alone or that the metamer-generating network encodes statistics in the metamer distribution that make it easier for the latter artificial agents to learn than for humans. Further investigation will be a valuable direction for future work. We've included a brief discussion of these points in our discussion (page 17).

2/ One analysis which the authors do not run (but which might shed light on this issue) is to compare the performance of networks meta-trained exclusively on metamer distributions but evaluated separately on rules and metamers. Presumably here the strong prediction from the authors' claim [that the networks are just learning the statistics and not the rules] is that performance would not differ?

The reviewer brings up a great point here. We ran this experiment (results below) and found that, when trained on metamers the agent typically still often does better on metamer tasks, in-line with our hypothesis that metamer tasks are often easier for agents than abstract tasks whereas abstract tasks are often easier for humans.

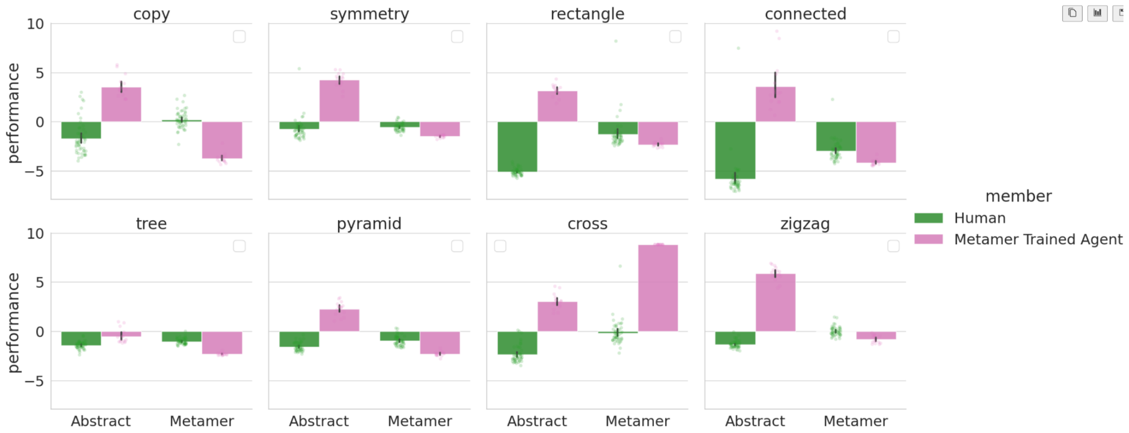

3/ Also the networks trained on abstract tasks really just do very badly – z > 5 here implies that every one of 10^5 games was performed worse than the nearest-neighbour random rule. It might be worth explaining why this is? In fact networks do so badly that it's actually quite difficult to see that people are doing better on abstract rules, which is what you might expect.

We agree that the networks trained on abstract tasks can sometimes generalize quite poorly on held-out abstract tasks. We would like to note that the same architectures, when trained on respective metamer tasks and tested on held-out metamer tasks, typically perform quite well and often better than humans. Additionally, Fig. S5 shows that sometimes networks trained on abstract tasks perform better on the out-of-distribution metamer test set, despite never having been trained on metamers. Both results show us that these networks are not necessarily performing poorly due to being underpowered, but because they differ in *inductive bias* and encode the "wrong" (i.e. misaligned with the human inductive biases that generated the rule-based stimuli) information when learning these tasks – the central theme of this work. We have included a discussion of these points in the discussion section (page 17).

4/ we don't know what the human prior is but presumably it is neither fully based on rules or statistics but a mixture of both. I wonder if the authors could have used different admixtures of rule-based and metamer stimuli in the meta-training set to try to infer this prior? Presumably networks meta-trained exclusively on rule-based stimuli will fail badly on the metamer task (as they largely do on the abstract task) but those meta-trained on just metamers have no reason to do better on the abstract task. But presumably, there is a sampling bias in the human experience of rules that tilts them towards doing better in the abstract task: could the authors re-create this using meta-training (this would be more similar to the Lake work the authors cite).

This is a great suggestion. First, we note that we hadn't actually trained any agents on mixtures. Before examining mixtures of rules as well as metamers, we first train on a mixture of all eight rules by randomly sampling from each rule during training. We then test on examples from each of the eight rules and their metamers (results below). We, perhaps surprisingly, found that this network consistently does better at the respective metamer tasks than the abstract tasks. Note that this network was never exposed to any metamer tasks during training, yet generalizes better on the metamer test set than held-out abstract tasks.

This result suggests a stark difference in inductive biases between the artificial agents and humans. We agree with the reviewer that the human inductive bias toward rules could be possible to meta-learn with enough exposure to rule-based stimuli, but jointly meta-training on all the rules we implemented did not impart a data-driven inductive bias toward rule-based

stimuli that humans seem to have. We suspect this would require a much more extensive sampling of rules and much broader meta-training, which is out of scope for this work. We have included these results and a discussion in the updated manuscript (FIg. S6 and page 14).

Note that we could also have used a more "blocked" scheme for training on all the rules vs the interleaved scheme we employ in this analysis. There is evidence that humans learn concepts better on blocked training schemes than interleaved schemes, but there is also evidence that typically neural networks perform better on interleaved schemes due to suffering from catastrophic forgetting on blocked schemes (Flesch et al. 2023a). Thus, an artificial agent that can learn the abstract tasks in our work in a human-like manner may require both a blocked training scheme and an architecture that supports continual learning (Flesch et al. 2023b). This may be an exciting direction for future work.

References:

Flesch, T., Saxe, A., & Summerfield, C. (2023). Continual task learning in natural and artificial agents. *Trends in Neurosciences*.

Flesch, T., Nagy, D. G., Saxe, A., & Summerfield, C. (2023). Modeling continual learning in humans with Hebbian context gating and exponentially decaying task signals. *PLOS Computational Biology*, *19*(1), e1010808.

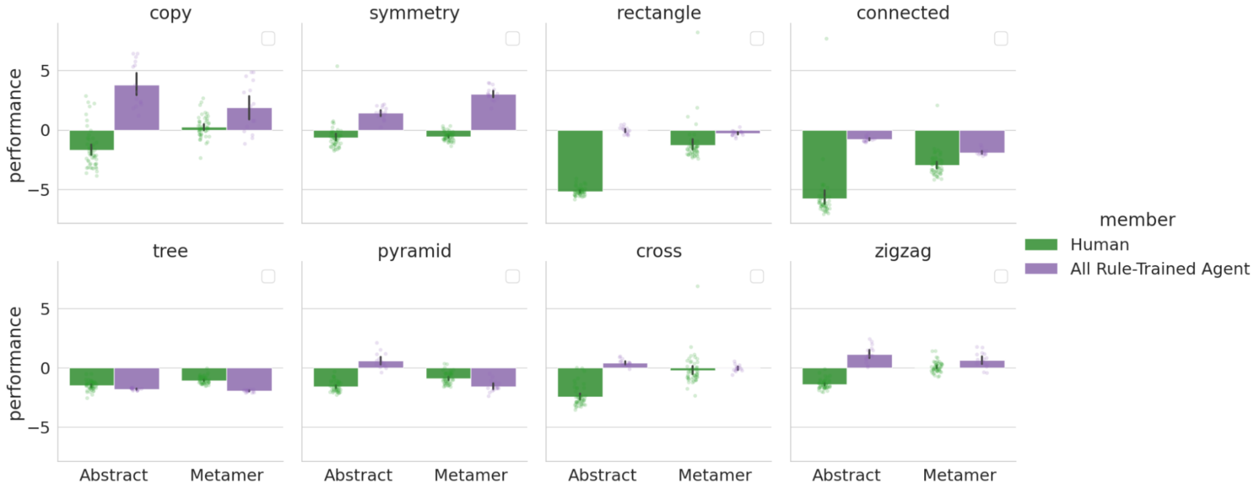

5/ Finally, does cross-training on different rules help? E.g. does network performance vary on the symmetry task if they are meta-trained on other rules / metamers in parallel? The other rules may contain information that is relevant (e.g. that rule-based patterns tend to obey Gestalt properties such as good continuity) that the network can use at evaluation (conditioning in the red tiles it has revealed so far). Presumably this is more similar to the task that humans face, given that they do not know which of the many possible rules they have encountered in the past might be relevant in the test phase.

We thank the reviewer for this interesting suggestion. We tried co-training on the different rules together in the previous section. Learning all the rules jointly did not give a big boost in performance. To look into in greater detail, below we report a heatmap of performance where rows correspond to training rules and columns correspond to test rules. We did this separately for metamers and abstract tasks. One interesting finding from this map is that all of the training rules produce comparable performance on the copy task, which may suggest the difficulty in learning the unique structure that the copy task employs. Another interesting finding is that training on zigzag metamers leads to good performance on all other metamer tasks, suggesting

that the statistics within the zigzag metamers helps the network learn all the other metamer tasks. We have included these results in the supplementary figures (Fig. S8). Additionally, our previous response shows results when the artificial agent is trained on a mixture of *all* the rules (included in the supplement as Fig. S6), which provides further evidence that the artificial agent is not able to learn the same general-purpose abstract knowledge that allows for generalization across rules that humans have.

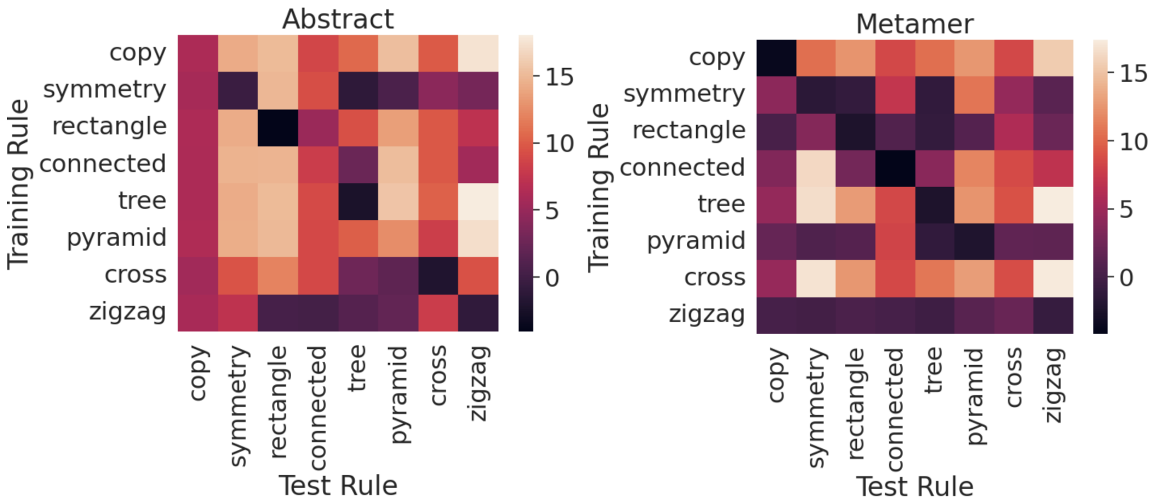

Reviewer #2: Stimuli that are generated based on some rule are inherently biased in terms of statistical properties. Successful generalization to held-out stimuli is therefore possible, to some extent, simply based on statistical learning even in the absence of any abstract reasoning (identification of the rule). This concern is particularly strong with AI agents that are trained on a very large number of samples and are known to excel at statistical learning. Here, the authors propose to use metamers, i.e. stimuli that are matched with rule-based stimuli in terms of statistics, and that would therefore be treated in just the same way as rule-based stimuli by an agent that relies on statistics (rather than the identification of the rule). The design of metamers stimuli that are meaningful to that purpose is a key element of this enterprise. The authors present a memater generation strategy in which the statistics that characterize rule-based stimuli are estimated by training a network. Human behavior was markedly different between rule-based stimuli and those statistically matched metamers. By contrast, the behavior of AI agents (different network architectures are tested) was much more similar. This difference indicates that human subjects have internalized (most of) the rules, and that AI agent have internalized only their statistical properties.

The question of what drives the ability to generalize is fascinating. The authors make an interesting methodological proposal. My main concerns are about the specific ways those metamers are created and used. The behavior of human and simulated agents is very interesting, and indicates that they rely on different processes (beyond my concerns about the way metamers were designed). Here are my main concerns.

1) The metamer task is not needed to distinguish between humans and AI agents here: there are clear differences in their behavior. The text sometimes suggests that it is difficult to conclude that the AI agents have not internalized the rule when tested on held-out samples generated by the rule (e.g. p. 6-7: "… examining average performance on held-out examples from structured task distributions. This approach may not reliability distinguish whether a system has truly internalized the underlying abstraction or whether it has learned statistical patterns"). However, during test, it is clear that the AI agents have not internalized the rule, in contrast to humans

(e.g. the beautiful examples of Fig 3B). The metamers are presented as being useful in order to test the hypothesis that AI agents have internalized statistics, but is not the internalization of statistics the only alternative to the internalization of rules? Surely, the AI agents have internalized something, because their behavior show clear evidence for some form of structure. Therefore it seems that rule-based held-out stimuli are enough to conclude that they have internalized statistics. If not true, then the authors should explain what could be the alternative(s).

We agree with the reviewer that the networks typically perform poorly at held-out rule-based stimuli. However, from such results, one might reach one of two conclusions – either the network architectures are underpowered (i.e. don't have the expressivity necessary to capture such distributions) or that they encode a different inductive bias. Our experimental design allows us to better isolate these possibilities. These same architectures, when trained on metamer tasks and tested on held-out metamer tasks, typically perform quite well and often better than humans. Additionally, Fig. S5 shows that sometimes networks trained on abstract tasks can generalize better to the metamer test set, despite never having been trained on metamers. Both results show us that these networks are not necessarily performing poorly due to being underpowered, but because they differ in *inductive bias* – the central theme of this work. This is a conclusion that can be uniquely revealed through analyzing human and machine performance on both abstract and metamer tasks. We have included a discussion of this point in the discussion section (page 17).

2) Let's assume that metamers are needed to prove that some agent has internalized some statistics rather than the rule (but see my point 1), perhaps a more informative use of metamers could be to reveal what type of statistics they have internalized. Different classes of metamers could correspond to different classes of statistics. It would be interesting to identify which statistics make stimuli metamers from the view point of the agent. This is not the approach considered by the authors, but it could extend the present work in very interesting ways.

We agree with the reviewer that this is a potentially interesting direction. As an initial exploration of this direction, below we report a heatmap of test-set performance where rows correspond to metamer training rules and columns correspond to metamer test performance. One finding we can extract from these data is that training on the zigzag metamer tasks yields good performance on all other metamer test rules, suggesting that the zigzag metamer tasks may encode certain qualitatively different statistics that help the model perform better at all other metamer tasks. It will be interesting to further explore this in future work. The below results have been included in the supplementary material (Fig. S8).

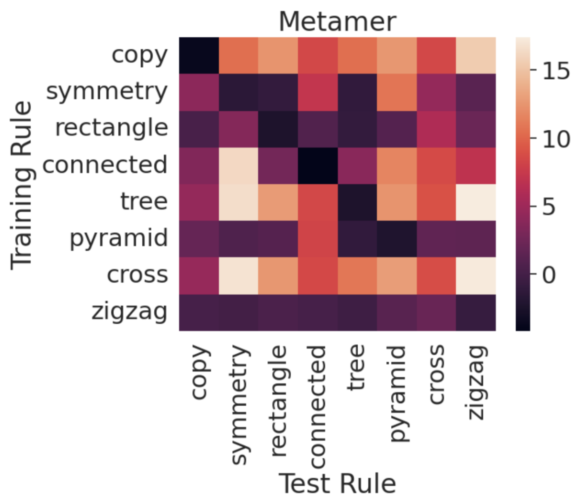

3) I don't fully understand why the metamers were designed in the specific way described by the authors. The statistical distribution that is learned (for each rule) is the conditional probability of each tile given full information about the other tiles. I have several questions/remarks.

We would first like to emphasize that the metamer generation process and the RL task we have humans and agents play are independent of each other. The former is being used as a tool to generate boards that contain various structures. The tile-revealing task is just a task designed atop these boards such that performance on the task tracks whether or not the board structure has been internalized by the RL agent trained on that board distribution. The metamer generating neural model is trained on conditionals simply because that allows us to perform Gibbs sampling and generate new samples from the newly learned distribution. The downstream RL agent also uncovers tiles sequentially, but this similarity to the Gibbs sampling process is purely incidental. We have included this point in the manuscript (page 7-8).

- First, such a distribution is relevant for the last step of the game, when all tiles but one have been revealed. This is a board configuration that subjects probably almost never see (because many tiles remain blank). The stimuli therefore seem to be matched for statistics that subjects are barely exposed to.

Because the tile-revealing task and the generation of metamer tasks are independent of each other, it is not necessary to use the statistics that subjects are exposed to during the tile-revealing task for the metamer generation network. The conditionals we used to generate the metamers serve as a convenient way to sample from a neural network's prior once exposed to a distribution (i.e. the rule-based grids). We've further emphasized this point in page 7-8 of the manuscript.

- Second, most conditionals actually do not comply with the rules (i.e. the board configuration on which the probability is conditioned on is impossible given the rule). Therefore, such conditionals could not be learned from actual exposure even by an optimal learner, simply because they are never encountered. I imagine that such "never encountered" conditionals should remain at some prior value, possibly a constant value, in an optimal learner. What do these "never encountered" conditionals look like in the trained network? Do the existence and value of such "never encountered" conditionals bias the metamer generation process in some way?

We agree that in an optimal learner, the never-encountered conditionals may not be the same ones learned by our metamer generating network. However, the purpose of this process is to generate a grid distribution similar to the rule-based grids but can reveal priors of neural

networks. Therefore, we expressly want the network to interpolate these “never-encountered conditionals” using its priors/biases. We have further emphasized this point in page 7-8 of the manuscript.

The reason we want to do this is because the network in the RL task will face the same problem of interpolating based on experience of sampled boards. We thus want to allow the network to exhibit similar biases as the RL agent in order to investigate the biases of the RL agent as compared to those of humans.

- Third, I don’t understand why an approach with network training is used in the first place. These conditional probabilities (of board configurations compatible with the rule) can all be evaluated analytically when the rule is given. To illustrate, for the rectangle configuration shown in Fig 2A, the conditional probability of the hidden tile being red is necessarily equal to one. For shapes with more degrees of freedom, these conditionals may not all be 1 or 0 but they can nevertheless be computed (e.g. for the tile at the tip of some cross shape, there may be uncertainty about the length of the branch resulting it a probability that is not 0 or 1). In this approach, the “never encountered” conditional probabilities (i.e. the ones incompatible with the rule) should have some default prior value. It therefore seems possible to run the same Gibbs sampling procedure but with the optimal conditionals under a given rule.

This is an interesting suggestion, but we believe that we can’t really do this for the following reason. In Gibbs sampling, we have to condition on some part of the data. We can either condition on the entire image (like we have done here) or condition on a single step of the rule. When conditioning based on the whole image, it is necessary to interpolate certain conditionals since the empirical distribution (from the true rule-based boards) is undersampled. It does not specify all possible conditionals (i.e. the “never-encountered” conditionals the reviewer mentions). If we condition the boards via the rules rather than the whole image, then that would be equivalent to generating from the rules. The goal of the metamer generating process is to generate a distribution of boards that is similar to the rule-based boards but ultimately different enough to reveal differences in behavior between humans and machines.

- Last, I am wondering whether 20 sweeps of Gibbs sampling are enough. To illustrate, the example rectangle metamers shown seem really different from a rectangle, in comparison some example connected metamers appear close to the rule. Would the rectangle metamers become more like rectangle with more sweeps? (the same concern of early stopping of the Gibbs sampler is true to other shapes)

We appreciate the concern raised by the reviewer. The number of sweeps needed for this process to converge can be approximately determined theoretically using the results of Graham & Morrison (1990), where they determined random walks on n-dimensional hypercubes can reach its stationary distribution for a number of steps of $N \gg \frac{1}{4} n \log n$. The right hand side is around 20 steps for our case of $n = 49$. Since a sweep is a total of 49 steps (one for each tile), 20 sweeps would be well above this threshold (980 steps). We have included this detail in our Methods section (page 19).

As further empirical qualitative evidence for this question, we ran the Gibbs Sampler for 100 sweeps rather than 20 sweeps. The results did not yield qualitatively different results (shown below).

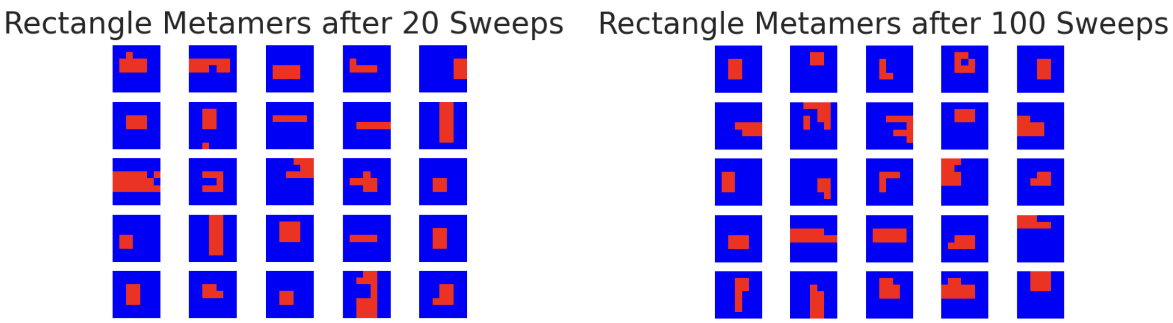

References:

Graham, R. L., & Morrison, J. A. (1990). Asymptotic Analysis of a Random Walk on a Hypercube with Alany Dimensions. *Random Structures and Algorithms*, *1*(1).

- The procedure used seems appropriate to match the low-order statistics (up to order 2, and perhaps even 3, Fig 2C), but is this enough? Why stopping at order 3?

The statistics used in Fig. 2 were employed to demonstrate that the two distributions had some similarity using some kind of objective and intuitive measure. The purpose of this is to contrast this similarity with the differences between human vs agent performance on the two distributions, which show that the subjective difficulty of the two distributions vary a lot between humans and agents. There are many ways to measure similarity between these grid stimuli, and this is just one such methodology. We used this particular statistic more as a sanity check that the grids are similar according to some objective metric. We do not believe that this statistic aptly characterizes how humans are processing these grids.

We could analyze at higher orders, but the particular matching statistic we used becomes more "degenerate" at higher orders. This is because the probability that one has a match at higher order (e.g. at order 4, a tile, its neighbor, its neighbor's neighbor, and its neighbor's neighbor's neighbor all have to match) will become increasingly less likely. Therefore, this particular matching statistic is not the best one to use to measure higher-order statistics of the boards. We added this point in page 8 of the manuscript.

Other remarks:
- my understanding is that a generative process characterizes a set or sequence of observations, but not a task, which is more about some cost function (what the agent is asked to do). Therefore, I find the notion of "task distribution" a bit confusing: the distributions referred are really about stimuli, not tasks.

We agree with the reviewer that the metamer generative process refers to the generation of grid stimuli. Our tile revealing task can be parametrized by a single grid stimulus, because which tiles are red in the underlying stimulus governs what the overall cost function of the task is (e.g. the mapping from actions to rewards). We hope this clarifies why we refer to the grids as a "task distribution" since each grid stimulus uniquely parametrizes a task/its associated cost function. We added a mention of this point in page 5 of the manuscript.

- the following paper on a potential mental language for geometrical shapes seems relevant: Sable Meyer et al, "A language of thought for the mental representation of geometric shapes", Cognitive Psychology, 2022
- the following paper on the coexistence of rules and statistics in humans also seem relevant: Maheu et al, "Rational arbitration between statistics and rules in human sequence processing", Nature Human Behavior, 2022. This paper stress that the inference of rules and statistics have

different learning dynamics: it is sometimes possible to infer a rule based on a few samples, whereas learning statistics necessarily require many samples. Learning dynamics could thus be informative about what is internalized: if high performance is achieved after a very limited number of samples, it can hardly be due to statistical learning, but more probably to the inference of a rule.

We have included these citations in the manuscript.

- Fig 3B. Great figure, but why not keeping the same red and blue hues as in the top panel? I was confused by the change in colors.

We have included this change in the new manuscript.
